# A multi-isotope (δ^13^C, δ^15^N, δ^34^S, δ^2^H) approach to establishing migratory connectivity in lesser snow geese: Tracking an overabundant species

**DOI:** 10.1371/journal.pone.0203077

**Published:** 2018-08-24

**Authors:** Drew N. Fowler, Elisabeth B. Webb, Frank B. Baldwin, Mark P. Vrtiska, Keith A. Hobson

**Affiliations:** 1 Missouri Cooperative Fish and Wildlife Research Unit, School of Natural Resources, University of Missouri, Columbia, Missouri, United States of America; 2 U. S. Geological Survey, Missouri Cooperative Fisheries and Wildlife Research Unit, School of Natural Resources, University of Missouri, Columbia, Missouri, United States of America; 3 Department of Sustainable Development, Province of Manitoba, Winnipeg, Manitoba, Canada; 4 Nebraska Game and Parks Commission, Lincoln, Nebraska, United States of America; 5 Department of Biology, Western University, London, Ontario, Canada; Stockholms Universitet, SWEDEN

## Abstract

Expanding populations of North American midcontinent lesser snow geese (*Anser caerulescens caerulescens*) have potential to alter ecosystems throughout the Arctic and subarctic where they breed. Efforts to understand origins of harvested lesser snow geese to better inform management decisions have traditionally required mark-recapture approaches, while aerial photographic surveys have typically been used to identify breeding distributions. As a potential alternative, isotopic patterns that are metabolically fixed within newly grown flight feathers following summer molting could provide inferences regarding geographic breeding origin of individuals, without the need for prior capture. Our objective was to assess potential to use four stable isotopes (δ^13^C, δ^15^N, δ^34^S, δ^2^H) from feather material to determine breeding origins. We obtained newly grown flight feathers from individuals during summer banding at three Arctic and two subarctic breeding colonies in 2014 (*n* = 56) and 2016 (*n* = 45). We used linear discriminant analyses to predict breeding origins from models using combinations of stable isotopes as predictors and evaluated model accuracy when predicting colony, subregion, or subpopulation levels. We found a strong inverse relationship between δ^2^H values and increasing latitude (*R*^2^ = 0.83), resulting in differences (*F*_4, 51_ = 90.41, *P* < 0.0001) among sampled colonies. No differences in δ^13^C or δ^15^N were detected among colonies, although δ^34^S in Akimiski Island, Baffin Island, and Karrak Lake were more enriched (*F*_4, 51_ = 11.25, *P* < 0.0001). Using δ^2^H values as a predictor, discriminant analyses improved accuracy in classification level as precision decreased [model accuracy = 67% (colony), 88% (subregion), 94% (subpopulation)]. Application of the isotopic methods we describe could be used to provide an alternative monitoring method of population metrics, such as overall breeding population distribution, region-specific productivity and migratory connectivity that are informative to management decision makers and provide insight into cross-seasonal effects that may influence migratory behavior.

## Introduction

North American midcontinent lesser snow geese (*Anser caerulescens caerulescens*) are long-distant migrants that predominately winter in temperate Arkansas and coastal Texas and Louisiana along the Gulf of Mexico [1–2]. Breeding distribution of midcontinent lesser snow geese ranges from 52° N to 85° N and spans an approximate 33° range in longitude. The extensive breeding range of lesser snow goose has resulted in numerous dispersed breeding colonies and the classification of northern and southern subpopulations (henceforth referred to as *Arctic* and *subarctic* subpopulations). Midcontinent lesser snow geese have been considered an overabundant species since 1999, based on explosive population growth facilitated by increasing agricultural production [3–4]. Efforts to understand the life history of lesser snow geese and their response to population reduction actions have been multifaceted; however, management actions taken to reduce the population have been deemed largely unsuccessful [5–6]. As the midcontinent population persists above desired levels, continued monitoring of metrics such as productivity, immigration / emigration rates among colonies, links between breeding and non-breeding areas (i.e., migratory connectivity) [7–8] and harvest demographics are important for tracking population change and informing future management decisions [5].

Estimation of age-specific annual survival and harvest rates among colony or subpopulation groups are important monitoring metrics and rely on long-term, annual banding efforts, in conjunction with band-recovery analysis [9]. To determine subpopulation affiliation, capture and marking of individuals must occur on the breeding grounds. Due to the large geographic breeding range of the population, general inaccessibility of the region, and relatively short molting period (i.e., when mass capture is possible), banding occurs at 5 distinct regions in the *Arctic* and *subarctic*, and indices of productivity (age ratios) at this time are assumed to be representative of the mid-continent population [10]. As an alternative, differentiation in stable isotope ratios across the wide geographic breeding range of lesser snow geese may provide a method to identify subpopulation or colony association of individuals of unknown origin, and provide annual indices of region-specific productivity that are likely less biased than those currently derived from the banded sample. Such a technique could also enable more frequent and cost-efficient assessments of breeding distribution, which have previously only been available through aerial photographic surveys.

Stable isotopes of light elements such as carbon (δ^13^C), nitrogen (δ^15^N), oxygen (δ^18^O), hydrogen (δ^2^H), and sulfur (δ^34^S) can serve as endogenous markers that allow inference to prior life history locations without requiring previous capture and recapture [11–12]. Briefly, food-webs differ regionally in isotopic signatures of light elements, and consumers incorporate and reflect tissue isotopic values specific to the food-web they inhabit [13]. These isotopic patterns can become fixed in metabolically inactive tissues such as feathers, claws, or hair [14] or exist in dynamic equilibrium in metabolically active tissues (e.g. blood, muscle) of an organism that moves among isotopically different food-webs [13, 15–16]. Therefore, isotopic analysis of select animal tissues has the capacity to reveal information on prior discrete events at both spatial and temporal scales.

Lesser snow geese undergo a complete flight feather molt in mid-summer, usually after breeding. Therefore, values of stable isotopes of newly grown flight feathers are representative of local source materials consumed at the time of flight feather growth (but see Fox et al. [17]). If individual breeding colonies produce unique feather isotopic signatures, then data conferring an individual’s previous year’s breeding / molt location (collected via harvest) could provide an alternative means of elucidating migratory connectivity and metrics of spatial population productivity. For example, Hobson et al. [18] used δ^2^H from flight feathers of hunter-harvested lesser scaup (*Aytha affinis*) to identify variation in breeding productivity of unique geographic regions. Similarly, isotopic analysis of muscle tissue has proven particularly useful in lesser snow geese to identify geographic wintering locations [19]. Consequently, joint application of these methods could provide insight into cross-seasonal effects that may exist within or among species that influence migratory behavior [20–21]. For instance, Paxton and Moore [22] assessed δ^13^C and δ^2^H values of red blood cells and feathers, respectively, from black-and-white warblers (*Mniotilta varia*) and found that quality of available winter habitats established differential body condition and proximate cues for spring migration and influenced individual migration movements.

To test the utility of stable isotope ratios in predicting geographic breeding origin of lesser snow geese, we collected newly grown flight feathers of adult female lesser snow geese at three *Arctic* and two *subarctic* breeding colonies in 2014. We evaluated stable isotope ratios expected to vary across latitude (δ^2^H) [23], diet (δ^13^C and δ^15^N) [24] and proximity to marine coastal environments (δ^34^S) [25] to provide for a wide potential variation in isotopic differentiation across colonies.

## Materials and methods

### Collection

**Fig 1:**
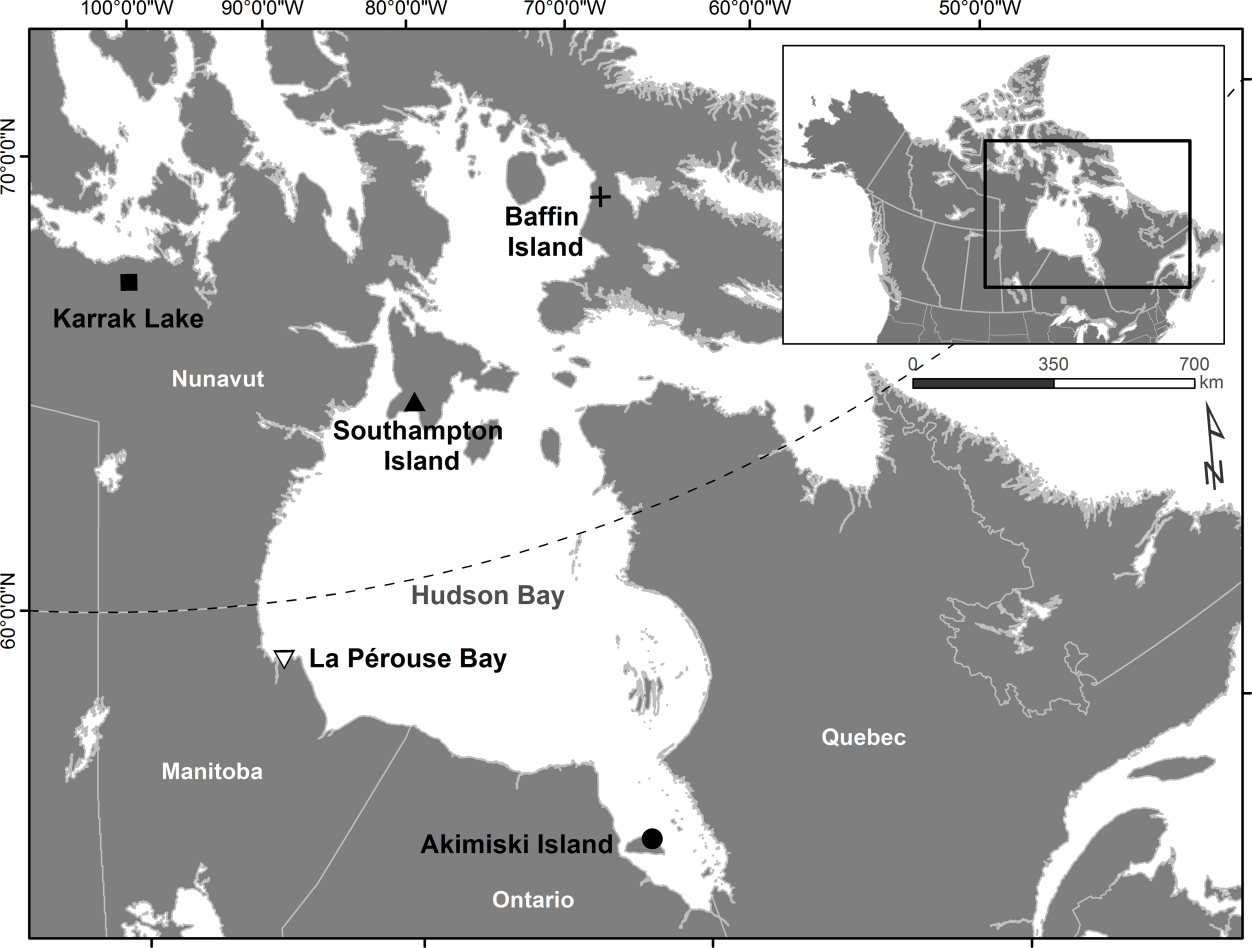
Location of sampled breeding colonies. Newly grown flight feathers of adult female (*n* = 56) midcontinent lesser snow geese (*Anser caerulescens caerulescens*) were collected for quantification of isotope signatures in July 2014. A second year of collections containing flight feathers from both adult males and females (*n* = 45) occurred at all colonies except Karrak Lake in July 2016.

To ensure certainty of isotope values originating from specific breeding locations, we collected newly grown flight feathers (P1 feather) of adult female lesser snow geese during routine mid-summer banding operations at three *Arctic* and two *subarctic* breeding colonies (Fig 1) in July 2014. *Arctic* breeding colonies included Southampton Island (63° 48' 30.67", -85° 41' 50.71") (*n* = 12), Baffin Island (66° 42' 46.04", -72° 33' 26.32") (*n* = 8) and Karrak Lake (67° 15' 37.30", -100° 16' 25.10") (*n* = 7). *Subarctic* breeding colonies included Akimiski Island (53° 06' 17.42", -80° 57' 28.73") (*n* = 11) and La Pérouse Bay (58° 43' 5.62", -93° 53' 21.54") (*n* = 18) (Fig 1). Upon removal, feathers were stored dry in paper envelopes until processing. All feathers from breeding colonies were collected in accordance with Environment Canada Animal Care Committee permit number #16JL01. Whole specimens during spring migration were collected under the University of Missouri’s Animal Care and Use Committee permit number 8191 and the United States Fish and Wildlife Service scientific collection permit number MB47969B-1.

Collected feathers were cleaned of surface oils in chloroform:methanol (2:1 *v*/*v*) solvent rinse. Stable hydrogen isotope measurements were performed on H_2_ gas derived from high-temperature flash pyrolysis of 350 ± 20 μg feather subsamples from the distal section of feather vanes (packed in silver capsules) using continuous-flow isotope-ratio mass spectrometry. Briefly, pyrolytic combustion (1350°C) was on a reactor with glassy carbon chips under helium flow in a Eurovector (Milan, Italy– www.eurovector.it) elemental analyzer interfaced with an Isoprime (Manchester, UK) continuous flow isotope ratio mass spectrometer (IRMS). Estimates of the nonexchangeable H in samples were derived from two keratin hydrogen-isotope reference materials (CNS: -197 ‰; KHS: -54.1 ‰) following the comparative equilibration approach of Wasssenaar and Hobson [26]. All δ^2^H results are reported for nonexchangeable H in delta notation, in units of per mil (‰), and normalized on the Vienna Standard Mean Ocean Water (VSMOW) standard scale. Based on within-run replicate (*n* = 8) measurements of keratin reference materials, we estimated measurement error (SD) to be ~ ± 2 ‰.

For δ^13^C and δ^15^N analyses, 0.5–1.0 mg of feather material (packed in tin capsules) was combusted online using a Eurovector 3000 elemental analyzer. The resulting CO_2_ was separated by gas chromatography (GC) and introduced into a Nu Horizon (Nu Instruments, Wrexham, UK– www.nu-ins.com) triple-collector isotope-ratio mass-spectrometer via an open split and compared to a pure CO_2_ or N_2_ reference gas. Stable nitrogen (^15^N/^14^N) and carbon (^13^C/^12^C) isotope ratios were expressed in *δ* notation, as parts per thousand (‰) deviation from the primary standards, atmospheric AIR and Vienna Pee Dee Belemnite (VPDB). Using previously calibrated internal laboratory standards (powdered keratin [BWB II: *δ* ^13^C = -20.0*‰*, *δ*^15^N = -14.1‰ and gelatin: *δ* ^13^C = -13.6*‰*, *δ*^15^N = -4.7‰]) within-run (*n* = 5), precision for *δ*^15^N and *δ*^13^C measurements was ~ ± 0.15‰.

For δ^34^S analyses, 3500 ± 100 ug of feather material (packed in tin capsules) was combusted in a Vario Pyro Cube (Elementar, Langenselbold, Germany– www.elementar.de) elemental analyzer and the resulting SO_2_ gas was introduced into Isoprime IRMS. Our laboratory standard was BWB-3 keratin (δ^34^S = 13.2‰) and δ values were reported relative to the Canyon Diablo Triolite (CDT) standard. Measurement precision, based on within-run replicate measurements of the lab standard was ± 0.3*‰*.

### Isotopic segregation of breeding colonies

To determine whether feathers from different breeding areas were isotopically distinct, we calculated mean feather stable isotope values within each breeding colony and ran a multiple analysis of variance (MANOVA; α < 0.05). Feather values for all four isotopes were normally distributed (Shapiro test: all *P*’s > 0.05) and homoscedastic (Levene’s test: all *P*’s > 0.05). We used Tukey posthoc tests to determine differences among individual isotope means and specific breeding colonies. To describe spatial variation in feather stable isotope ratios across the geographic range of selected breeding colonies, we used simple linear regression analyses to relate stable isotope values to variation in latitude and longitude.

We used linear discriminant function analyses to develop a suite of candidate models to predict breeding colony association using combinations of δ^2^H, δ^13^C, δ^15^N, and δ^34^S values (Table 1). Based on MANOVA results, we developed a second set of linear discriminant models in which we grouped individuals from Southampton Island and Baffin Island together as a subregion based on their similar feather δ^2^H values (Table 1). Finally, we evaluated a set of linear discriminant models where colonies were compressed into either *Arctic* or *subarctic* subpopulation association (Table 1). We used K-fold cross validation (K = 5) to evaluate the accuracy of assignment for discriminant functions built to determine colony and subpopulation association using the “lda” function in the MASS package in Program R [27]. We determined the strength of individual models based on the percent accuracy of individuals from each breeding colony in 2014 whose isotopic value correctly represented their breeding colony [28].

**Table 1:** Models selected for linear discriminant function analysis.

Model	
Colony	~ δ^2^H + δ^13^C + δ^15^N + δ^34^S
Colony	~ δ^2^H + δ^34^S
Colony	~ δ^2^H
Subregion	~ δ^2^H + δ^34^S
Subregion	~ δ^2^H
Subpopulation	~ δ^2^H + δ^34^S
Subpopulation	~ δ^2^H

Models were developed to identify classifications at the individual colony level, subregion, and subpopulation level based on stable hydrogen (δ^2^H), nitrogen (δ^15^N), carbon (δ^13^C), and sulfur (δ^34^S) isotope values in feathers collected from adult female lesser snow geese at *Arctic* and *subarctic* breeding colonies during summer banding in 2014.

As a proof of concept, we collected flight feathers (*n* = 40) from adult, female snow geese harvested in Arkansas, Missouri, Nebraska, and South Dakota during spring migration 2015. In a preliminary investigation, feathers from geese of unknown origins were prepared in identical manner described above and, based on development of discriminant modeling results, analyzed for δ^2^H values. Unknown individuals were then assigned origin based on the greatest posterior probability of belonging to one of the five colonies (and subpopulation association) predicted by a linear discriminant model based on δ^2^H using the “lda” “predict” function in the MASS package.

### Inter-annual variability in δ^2^H

The use of feather δ^2^H values as a reliable marker of goose origin is dependent upon how well modeled food-web δ^2^H and corresponding feather δ^2^H (δ^2^H_f_) matches predictions. Such models are ultimately based on the long-term dataset provided by the International Atomic Energy Association (IAEA) Global Network of Isotopes in Precipitation (GNIP). Predicted local precipitation values are derived from kriged data using actual amount-weighted monthly average precipitation δ^2^H (δ^2^H_p_) values from a network of sampling stations. Those data are then typically transformed into expected δ^2^H_f_ values for spatially explicit assignment. Here we relied instead on describing expected feather isotope values based solely on year-specific, ground-truthed sampling of feathers. For δ^2^H_f_, some interannual variation can be expected [12, 29–30] and such uncertainty is propagated in most spatially explicit probabilistic assignments [31]. To estimate potential interannual variation in lesser snow goose δ^2^H_f_, we obtained additional lesser snow goose feathers from known colonies in 2016. Here, secondary covert flight feathers from adult male and female birds were collected at Akimiski Island (*n* = 9), La Pérouse Bay (*n* = 10), Baffin Island (*n* = 11) and Southampton Island (*n* = 15) in 2016 and analyzed for δ^2^H_f_. We compared differences in δ^2^H_f_ by colony, year, and the interaction with a two-way analysis of variance (α = 0.05). We created a discriminant function model using δ^2^H_f_ values based on feathers collected in 2016 and used feathers derived from the same colonies in 2014 as a test group. Similarly, we used the δ^2^H_f_ discriminant function model based on 2014 feathers to obtain model predictions of the known 2016 feathers and compare differences in error rate between the two years. We used R 3.3.3 (R Core Development Team 2017) for all statistical analyses.

## Results

### Comparison of isotopes by breeding colonies

**Fig 2:**
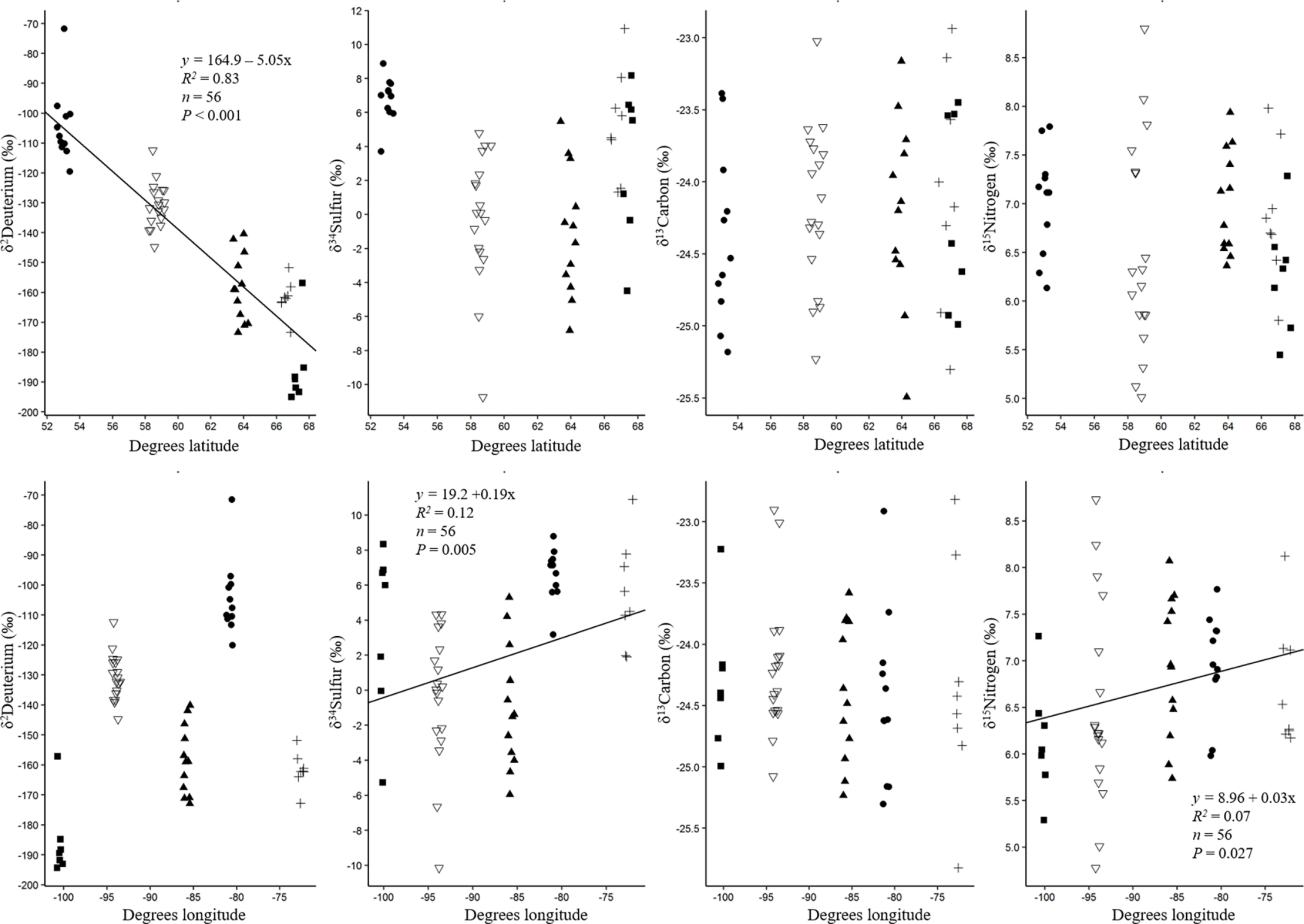
Relationships between isotopes (δ^2^H, δ^34^S, δ^13^C, and δ^15^N) analyzed from feather material to latitude and longitude for adult lesser snow geese collected July, 2014. The top panel presents relationships between isotope values and latitude. The bottom panel presents relationships between isotope values and longitude. Breeding colonies are Akimiski Island (closed circle), La Pérouse Bay (open triangle), Southampton Island (closed triangles), Baffin Island (crosses), and Karrak Lake (closed squares). Linear regression lines are provided only for slopes that were significantly (*P* < 0.05) different from zero.

Simple linear regression analysis indicated a strong inverse relationship between δ^2^H_f_ values and increasing latitude (*R*^2^ = 0.83; Fig 2). No other isotope showed any detectable relationship between δ values and latitude. Minor relationships between δ^34^S and δ^15^N and longitude existed as depletion increased slightly in more westerly colonies, although variation in the data explained by the relationship was low (*R*^2^ = 0.12, *R*^2^ = 0.07, respectively; Fig 2).

Results from the MANOVA indicated overall differences (*F*_16, 204_ = 7.51, *P* < 0.0001) in measured isotope values among breeding colonies for feathers of known origin collected in 2014 (Table 2). However, only δ^2^H_f_ and δ^34^S differed among colonies (δ^2^H_f_: *F*_4, 51_ = 90.41, *P* < 0.0001; δ^34^S: *F*_4, 51_ = 11.25, *P* < 0.0001) whereas δ^13^C and δ^15^N did not (δ^13^C: *F*_4, 51_ = 0.29, *P* = .8818; δ^15^N: *F*_4, 51_ = 2.02, *P* = 0.1047). Post hoc Tukey tests indicated δ^2^H_f_ differed separately among Akimiski Island, La Pérouse Bay, and Karrak Lake. Feather deuterium values did not differ between Southampton Island and Baffin Island but were different from remaining colonies (Table 2). Values of feather δ^34^S did not differ among Akimiski Island, Baffin Island, and Karrak Lake but as a group were more enriched than values found at La Pérouse Bay and Southampton Island (Table 2).

**Table 2:** Mean and SE of stable hydrogen (δ^2^H), nitrogen (δ^15^N), carbon (δ^13^C), and sulfur (δ^34^S) isotope values in feathers collected from adult female lesser snow geese at Arctic and subarctic breeding colonies during summer banding in 2014^a^.

Sampled Colonies	Subpopulation	δ^2^H (‰)	δ^15^N (‰)	δ^13^C (‰)	δ^34^S (‰)
Akimiski Island (*n* = 11)	Subarctic	-104.17 (3.84) A	6.98 (0.10) A	-24.40 (0.18) A	6.68 (0.39) A
La Pérouse Bay (*n* = 18)	Subarctic	-130.81 (1.77) B	6.44 (0.23) A	-24.21 (0.09) A	-0.33 (0.91) B
Southampton Island (*n* = 12)	Arctic	-158.41 (3.30) C	6.99 (0.18) A	-24.36 (0.15) A	-0.98 (1.07) B
Baffin Island (*n* = 8)	Arctic	-161.85 (2.10) C	6.84 (0.23) A	-24.24 (0.25) A	5.37 (1.12) A
Karrak Lake (*n* = 7)	Arctic	-185.54 (4.98) D	6.31 (0.25) A	-24.26 (0.24) A	3.41 (1.76) A

^a^ Means with the same letters within a column are not different (*P*>0.05).

**Fig 3:**
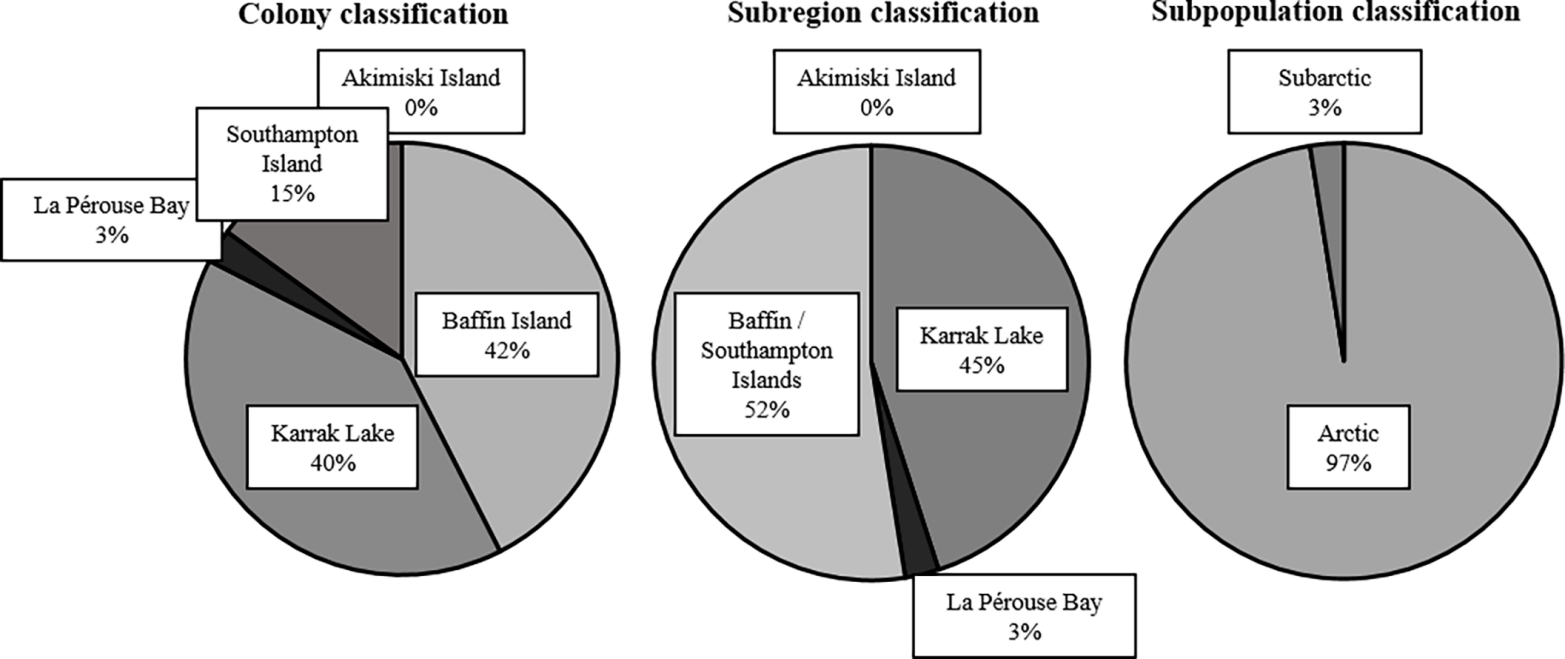
Breeding origin classification percentages from isotopic signatures of lesser snow geese (*n* = 40) harvested during spring migration, 2015.

Values of δ^2^H_f_ and δ^34^S most accurately predicted breeding colony association, when using K-fold cross-validation, compared to the comprehensive model using δ^2^H_f_, δ^13^C, δ^15^N, and δ^34^S or δ^2^H_f_ alone (overall model accuracy of 80%, 76%, and 67%, respectively; Table 3). When predicting at the colony level, misclassification was greatest between Baffin Island and Southampton Island (Table 3). However, grouping Baffin Island and Southampton Island as a subregion improved overall model accuracies. Prediction for the model using δ^2^H_f_ alone was more accurate (88%) than the model collectively using δ^2^H_f_ and δ^34^S (86%; Table 3). Finally, greatest prediction accuracy was achieved when breeding colonies were classified according to subpopulation. The model using δ^2^H_f_ alone was sufficiently accurate to predict subpopulation association relative to the δ^2^H_f_ and δ^34^S model (94% accuracy compared with 93%; Table 3). Posterior classification probabilities using the δ^2^H_f_ model for 40 geese harvested during the 2015 spring migration increased as classification precision level decreased (S1 Table). Classifying individuals to either the subregion or subpopulation level incrementally reduced uncertainty in assignment probabilities without substantially changing the overall proportions of classifications to other groups (Fig 3).

**Table 3:** Prediction accuracies by discriminant function models, partitioned across the colony, subregion, and subpopulation level of adult lesser snow geese collected in 2014 using K-fold cross validation.

		**Colony Accuracy**				
**Model**	Overall model accuracy	Akimiski Island (*n* = 11)	La Pérouse Bay (*n* = 18)	Baffin Island (*n* = 8)	Southampton Island (*n* = 12)	Karrak Lake (*n* = 12)
δ^2^H + δ^13^C + δ^15^N + δ^34^S	76%	91%	89%	50%	58%	86%
δ^2^H + δ^34^S	80%	91%	94%	63%	67%	71%
δ^2^H	67%	91%	94%	0%	42%	86%
		**Subregion Accuracy**				
		Akimiski Island	La Pérouse Bay	Baffin / Southampton Islands (*n* = 20)		Karrak Lake
δ^2^H + δ^34^S	86%	82%	89%	85%		86%
δ^2^H	88%	91%	89%	85%		86%
		**Subpopulation Accuracy**				
		Subarctic (*n* = 29)		Arctic (*n* = 27)		
δ^2^H + δ^34^S	93%	97%		89%		
δ^2^H	94%	97%		93%		

### Inter-annual variability in δ^2^H_f_

Values of δ^2^H_f_ differed interactively among colonies and year (*F*_3, 864_ = 4.24, *P* = 0.0076) as only lesser snow geese collected at Akimiski Island indicated differences in δ^2^H_f_ values between 2014 and 2016 (Table 4). This annual difference in δ^2^H_f_ values at Akimiski Island was reflected by low Akimiski Island prediction accuracies (44%) when predictions of 2016 colony classification were made using the δ^2^H_f_ discriminant function derived from feathers collected in 2014 (Table 5). In contrast, δ^2^H_f_ discriminant functions derived from each respective year to predict subregion and subpopulation level increased overall prediction accuracies by 29% and 40%, respectively (Table 5).

**Table 4:** Comparison of mean and SE of stable hydrogen (δ^2^H) isotope values in feathers collected from adult lesser snow geese in 2014 and 2016^a^.

Sampled Colonies	Subpopulation	δ^2^H (‰)	
		2014	2016
Akimiski (2014, *n* = 11; 2016, *n* = 9)	Subarctic	-104.17 (3.84) A	-117.73 (1.72) B
La Pérouse Bay (2014, *n* = 18; 2016, *n* = 10)	Subarctic	-130.81 (1.77) A	-127.90 (0.78) A
Southampton (2014, *n* = 12; 2016, *n* = 15)	Arctic	-158.41 (3.30) A	-156.62 (1.64) A
Baffin (2014, *n* = 8; 2016, *n* = 11)	Arctic	-161.85 (2.10) A	-170.36 (4.73) A

^a^ Means with the same letters within a row are not different (*P*>0.05).

**Table 5:** Comparison of prediction accuracies of annually derived discriminant function models, partitioned across the colony, subregion, and subpopulation level of adult lesser snow geese collected in 2014 and 2016, using K-fold cross-validation.

			**Colony Accuracy**			
**Model**	**Prediction Group**	Overall model accuracy	Akimiski Island	La Pérouse Bay	Baffin Island	Southampton Island
δ^2^H Model 2014 (*n* = 49)	2016 Colony Feathers (*n* = 45)	69%	44%	100%	73%	60%
δ^2^H Model 2016 (*n* = 45)	2014 Colony Feathers (*n* = 49)	69%	100%	83%	38%	42%
			**Subregion Accuracy**			
			Akimiski Island	La Pérouse Bay	Baffin / Southampton Islands	
δ^2^H Model 2014 (*n* = 49)	2016 Colony Feathers (*n* = 45)	89%	44%	100%	100%	
δ^2^H Model 2016 (*n* = 45)	2014 Colony Feathers (*n* = 49)	90%	100%	83%	90%	
			**Subpopulation Accuracy**			
			Subarctic		Arctic	
δ^2^H Model 2014 (*n* = 49)	2016 Colony Feathers (*n* = 45)	100%	100%		100%	
δ^2^H Model 2016 (*n* = 45)	2014 Colony Feathers (*n* = 49)	94%	97%		90%	

## Discussion

The primary goal of this study was to evaluate potential to determine breeding origins of lesser snow geese using a suite of stable isotopes analyzed from feather material and evaluate the combination of spatial precision and accuracy achievable under various origin scales. We found that among the four stable isotopes tested (δ^2^H, δ^13^C, δ^15^N, and δ^34^S), deuterium (δ^2^H) was the most distinct across the sampled breeding colonies. Generally, deuterium values were more depleted with increasing latitude. The positive relationship between depleted precipitation deuterium and increasing latitude, and the positive relationship between precipitation deuterium and feather deuterium is well established [23–31]. However, variance in the latter relationship can vary among taxa [31]. Our study quantified deuterium specifically in feather material of a large-bodied migratory bird whose breeding range extends across a wide Arctic and subarctic latitude. In contrast, δ^13^C and δ^15^N values did not vary across sampled breeding colonies and their inclusion in linear discriminant analyses reduced overall prediction accuracy slightly. Lack of difference in δ^13^C and δ^15^N among colonies may be related to similar plant foraging material across breeding areas [6].

We found differences in δ^34^S values among two groups of colonies. Values of δ^34^S were more enriched in colonies at Akimiski Island, Baffin Island, and Karrak Lake, in contrast to more depleted values at La Pérouse Bay and Southampton Island. In general, δ^34^S values are expected to be enriched in breeding areas in proximity to coastal areas where prevailing winds transfer sulfates from sea spray onto land [32–33]. Additionally, δ^34^S in feathers could be enriched based on the predominant consumption of marine foods over terrestrial resources. For lesser snow geese breeding at La Pérouse Bay, coastal marsh degradation from heavy grazing forced the colony to shift more inland towards freshwater marshes [34]. This inland shift and utilization of freshwater wetlands may explain the depleted δ^34^S values we observed in feathers from lesser snow geese at La Pérouse Bay in this study. However, lesser snow geese breeding at higher latitudes are thought to rely more heavily on freshwater wetlands than southern breeders [35], yet δ^34^S values from individuals from these areas in our study were more enriched. Nonetheless, δ^34^S could be useful as a secondary discriminant predictor of breeding colonies whose classification is confounded using only δ^2^H_f_ because of similar values among colonies. This was the case for classification of individuals from Baffin Island and Southampton Island. Using feather δ^34^S in addition to δ^2^H_f_ values improved overall model prediction accuracy from 67% to 80%. However, while improving prediction between Baffin Island and Southampton Island, using δ^34^S in conjunction with δ^2^H_f_ increased misclassification rates for individuals originating from Karrak Lake.

Selecting to use a discriminant function analysis derived at the colony, subregion, or subpopulation level results in a tradeoff between accuracy and precision. Our discriminant functions based on δ^2^H_f_ that classified individuals at the subregion or subpopulation level were 34% and 40% more accurate compared to a model classifying individuals to the colony level; however, the spatial scope of inference was reduced. Using a discriminate function based on δ^2^H_f_ that classifies an individual to the subregion level may currently be the most appropriate balance between accuracy of classification and inferential spatial scope.

The ability to isotopically distinguish among origin of individuals, even at the scale of subregion, has several useful applications, but the most important may be an improved understanding of breeding distribution. Overall population size can be estimated from age-specific harvest rate estimates using Lincoln’s method [36] obtained from banding data and age-specific harvest estimates, which are obtained from long-established hunter harvest surveys in Canada and the USA wherein a portion of hunters submit tail and wing feathers. Lincoln abundance estimates have become an important metric for monitoring population status for some populations of North American geese [10]. The components used in Lincoln estimates are available over long time periods and are thought to be more reliable than other survey methods (e.g., mid-winter aerial surveys, aerial photo surveys) that may be biased to an unknown extent by issues related to detection, speciation, and sampling coverage. Although they account for an unknown proportion of the total population and are conducted infrequently, photo-surveys of known nesting colonies have been used to improve the understanding of the breeding distribution of this population [37]. Lincoln estimates have become the preferred metric for monitoring changes in overall abundance to avoid the need for costly and logistically challenging photo-surveys [10]. However, Lincoln estimates do not provide information about the distribution of the breeding population, thus identifying an alternative approach to estimating breeding distribution would be an important contribution to population management and allow for monitoring migratory connectivity. Isotopic classification of clipped primary feathers submitted by hunters through hunter harvest surveys would permit partitioning of Lincoln estimates, at least to the level of subpopulation. Our classification of forty adult migrants collected during spring migration illustrates the potential to provide an alternative index that describes spatial distribution of the population. An improved understanding of breeding distribution is of management interest because the relationship between adult survival and harvest differs between northern and southern nesting segments of the population [5]. Thus, course-scale population distribution should be a consideration when designing monitoring programs, or when determining where to establish or expand research on population-level management questions.

Another important monitoring metric available from annual banding programs is the development of a productivity index (i.e., age ratio at banding). However, the degree to which age ratios at banding reflect overall productivity could be influenced by local weather or predation pressure [38], age of young at capture (e.g., exposure days) [39], and individual decisions made by banders (e.g., targeting smaller brood flocks or large groups of non-breeders, removal of incomplete captures from calculations, focusing on the best areas of production). Determining isotopic values of a random sample of primary feathers submitted by hunters through the annual harvest survey would likely yield a more spatially representative index of annual production than can be provided by annual banding programs.

Predicting breeding origins of lesser snow geese sampled away from breeding colonies based on a discriminant analysis derived from δ^2^H_f_ values of feathers grown at known colonies constrains classifications only to those colonies where feathers have been analyzed and included in the analysis. One drawback to our approach is the potential to misclassify an individual to one of the known colonies when true origin was another colony not assessed in this study. Similarly, we primarily analyzed feathers from individuals who successfully bred and were molting in July. Because of logistical constraints, banding efforts at Southampton Island only target failed breeders or those who forego breeding altogether. Therefore, our models are primarily derived from one cohort of individuals, breeders, and do not account for individuals who initiate a subsequent migration elsewhere to molt and regrow feathers. Extensive detail regarding the destination of molt migrants from specific lesser snow goose colonies is limited. In one study on lesser snow geese at La Pérouse Bay, molt migrants moved north to the McConnell River, about 250 km away [40]. Additional research identifying additional molt migrant destinations from other colonies would assist ability to interpret isotopic feather values. Additionally, we used only adult feathers to derive our linear discriminant functions. Previous works in other avian guilds (passerines and raptors) have identified more depleted deuterium values in juveniles compared with adults from the same known origin, potentially thought to be influenced by differences in diet, feather growth rate and metabolism [41–43]. While we do not have reasons to suspect differences in diet among juvenile and adult lesser snow geese, evaluating differences in δ^2^H_f_ among age classes will be important to ensure predictive accuracy. We also observed some inter-annual variation of δ^2^H_f_ values at Akimiski Island between sampling in 2014 and 2016 that resulted in misclassification when using one year as a training set for a model and the other as a test set. These limitations emphasize the importance of continued multi-year sampling (of newly grown feathers during molt) and an increase in the number of colonies sampled. Data from additional sampling could be used to calibrate a functional based map of δ^2^H_f_ across the Canadian Arctic and result in increased accuracy and precision of lesser snow goose breeding origin classification using stable isotope analysis.

## Conclusions

Using isotopic markers found in metabolically active or inactive animal material provides a pathway to identify heterogeneous breeding origins within harvested populations without requiring previous capture, marking, and recapture. Our study provides an initial demonstration of the utility of identifying migratory connectivity through stable isotopes in lesser snow geese. Yet, our approach could be further developed through the calibration of the long-term GNIP dataset from lesser snow goose δ^2^H_f_ values, resulting in a species-specific breeding isoscape. The application of these data could be used to provide a more robust annual estimate of subregion or subpopulation size based on isotopic partitioning of harvest survey parts. As lesser snow geese have increased in abundance, juvenile-to-adult age ratios have declined [44] in response to increasing density-dependence [45]. Using previous techniques demonstrated to assess productivity [18] and migratory connectivity in harvested gamebirds [46] could be implemented in midcontinent lesser snow geese and facilitate comprehensive monitoring informative to future management decisions.

## Supporting information

S1 Dataset Stable isotope values of hydrogen (δ^2^H), nitrogen (δ^15^N), carbon (δ^13^C), and sulfur (δ^34^S) in feathers collected from adult female lesser snow geese (*n* = 99) at Arctic and subarctic breeding colonies during summer banding in July, 2014 and 2016. Stable isotope values are reported in per mil notation (‰). δ^15^N _,_ δ^13^C, and δ^34^S were not analyzed in feathers collected in 2016.
(DOCX)

S2 Dataset Stable isotope values of hydrogen (δ^2^H) in feathers collected from adult female lesser snow geese (*n* = 40) collected during spring migration in 2015. Stable isotope values are reported in per mil notation (‰).
(DOCX)

S1 Table Posterior probabilities of colony, subregion, and subpopulation association for lesser snow geese (*n =* 40) harvested during spring migration, 2015, based on linear discriminant analysis using δ^2^H_f_ from feathers collected on known breeding colonies in 2014 as a discriminating variable. Percentages in bold identify the greatest probability of colony and subpopulation association for each collected specimen.
(PDF)
