# Supplementary material for: A multi-isotope (δ13C, δ15N, δ34S, δ2H) approach to establishing migratory connectivity in lesser snow geese: Tracking an overabundant species
Source: PLoS One. 2018 Aug 24;13(8):e0203077. doi: 10.1371/journal.pone.0203077 (PMC6108521; doi:10.1371/journal.pone.0203077)
Supplement: S1 Dataset — Stable isotope values are reported in per mil notation (‰). δ15N , δ13C, and δ34S were not analyzed in feathers collected in 2016. (DOCX) [file pone.0203077.s001.docx]

| Individual | Collection Year | Collection Colony | Latitude | Longitude | Sex | Age | Feather | δ^2^H | δ^15^N | δ^13^C | δ^34^S |
| --- | --- | --- | --- | --- | --- | --- | --- | --- | --- | --- | --- |
| 1 | 2014 | Karrak Lake | 67.26036 | -100.27364 | F | AHY | Primary flight | -189.07 | 6.21 | -23.74 | 6.04 |
| 2 | 2014 | Karrak Lake | 67.26036 | -100.27364 | F | AHY | Primary flight | -184.72 | 7.60 | -25.05 | -4.78 |
| 3 | 2014 | Karrak Lake | 67.26036 | -100.27364 | F | AHY | Primary flight | -194.77 | 6.07 | -24.74 | 1.69 |
| 4 | 2014 | Karrak Lake | 67.26036 | -100.27364 | F | AHY | Primary flight | -191.84 | 6.43 | -24.55 | 6.59 |
| 5 | 2014 | Karrak Lake | 67.26036 | -100.27364 | F | AHY | Primary flight | -188.23 | 6.07 | -24.59 | -0.05 |
| 6 | 2014 | Karrak Lake | 67.26036 | -100.27364 | F | AHY | Primary flight | -193.46 | 6.37 | -23.75 | 8.19 |
| 7 | 2014 | Karrak Lake | 67.26036 | -100.27364 | F | AHY | Primary flight | -156.68 | 5.45 | -23.38 | 6.21 |
| 8 | 2014 | Southampton Island | 63.80852 | -85.69742 | F | AHY | Primary flight | -159.04 | 6.19 | -24.61 | 2.90 |
| 9 | 2014 | Southampton Island | 63.80852 | -85.69742 | F | AHY | Primary flight | -173.17 | 7.28 | -24.25 | -1.40 |
| 10 | 2014 | Southampton Island | 63.80852 | -85.69742 | F | AHY | Primary flight | -146.82 | 6.79 | -25.35 | 0.23 |
| 11 | 2014 | Southampton Island | 63.80852 | -85.69742 | F | AHY | Primary flight | -141.84 | 7.41 | -24.63 | -0.44 |
| 12 | 2014 | Southampton Island | 63.80852 | -85.69742 | F | AHY | Primary flight | -157.21 | 7.74 | -24.66 | -3.06 |
| 13 | 2014 | Southampton Island | 63.80852 | -85.69742 | F | AHY | Primary flight | -140.40 | 6.00 | -25.02 | -6.38 |
| 14 | 2014 | Southampton Island | 63.80852 | -85.69742 | F | AHY | Primary flight | -171.42 | 8.04 | -24.20 | -0.88 |
| 15 | 2014 | Southampton Island | 63.80852 | -85.69742 | F | AHY | Primary flight | -163.28 | 7.25 | -23.85 | -3.29 |
| 16 | 2014 | Southampton Island | 63.80852 | -85.69742 | F | AHY | Primary flight | -150.84 | 6.85 | -24.09 | 3.83 |
| 17 | 2014 | Southampton Island | 63.80852 | -85.69742 | F | AHY | Primary flight | -158.72 | 6.36 | -24.04 | -3.94 |
| 18 | 2014 | Southampton Island | 63.80852 | -85.69742 | F | AHY | Primary flight | -170.87 | 7.23 | -23.41 | -5.06 |
| 19 | 2014 | Southampton Island | 63.80852 | -85.69742 | F | AHY | Primary flight | -167.31 | 6.79 | -24.18 | 5.78 |
| 20 | 2014 | Akimiski Island | 53.10484 | -80.95798 | F | AHY | Primary flight | -71.26 | 7.05 | -24.82 | 6.62 |
| 21 | 2014 | Akimiski Island | 53.10484 | -80.95798 | F | AHY | Primary flight | -97.28 | 7.27 | -23.38 | 8.42 |
| 22 | 2014 | Akimiski Island | 53.10484 | -80.95798 | F | AHY | Primary flight | -100.60 | 7.40 | -25.07 | 7.35 |
| 23 | 2014 | Akimiski Island | 53.10484 | -80.95798 | F | AHY | Primary flight | -111.62 | 6.45 | -23.82 | 7.32 |
| 24 | 2014 | Akimiski Island | 53.10484 | -80.95798 | F | AHY | Primary flight | -109.83 | 7.11 | -24.60 | 6.00 |
| 25 | 2014 | Akimiski Island | 53.10484 | -80.95798 | F | AHY | Primary flight | -108.02 | 6.75 | -23.72 | 7.50 |
| 26 | 2014 | Akimiski Island | 53.10484 | -80.95798 | F | AHY | Primary flight | -99.84 | 7.49 | -24.92 | 6.32 |
| 27 | 2014 | Akimiski Island | 53.10484 | -80.95798 | F | AHY | Primary flight | -119.83 | 6.98 | -23.91 | 5.52 |
| 28 | 2014 | Akimiski Island | 53.10484 | -80.95798 | F | AHY | Primary flight | -109.97 | 6.95 | -25.06 | 7.39 |
| 29 | 2014 | Akimiski Island | 53.10484 | -80.95798 | F | AHY | Primary flight | -104.56 | 6.95 | -24.57 | 7.33 |
| 30 | 2014 | Akimiski Island | 53.10484 | -80.95798 | F | AHY | Primary flight | -113.01 | 6.41 | -24.56 | 3.67 |
| 31 | 2014 | La Pérouse Bay | 58.71823 | -93.88931 | F | AHY | Primary flight | -138.75 | 7.42 | -24.03 | 1.37 |
| 32 | 2014 | La Pérouse Bay | 58.71823 | -93.88931 | F | AHY | Primary flight | -138.12 | 6.09 | -23.47 | -0.35 |
| 33 | 2014 | La Pérouse Bay | 58.71823 | -93.88931 | F | AHY | Primary flight | -120.92 | 7.59 | -24.52 | -6.25 |
| 34 | 2014 | La Pérouse Bay | 58.71823 | -93.88931 | F | AHY | Primary flight | -138.92 | 6.34 | -24.12 | -10.48 |
| 35 | 2014 | La Pérouse Bay | 58.71823 | -93.88931 | F | AHY | Primary flight | -129.68 | 5.12 | -24.30 | -3.38 |
| 36 | 2014 | La Pérouse Bay | 58.71823 | -93.88931 | F | AHY | Primary flight | -130.64 | 5.80 | -24.05 | -2.03 |
| 37 | 2014 | La Pérouse Bay | 58.71823 | -93.88931 | F | AHY | Primary flight | -126.36 | 5.47 | -23.96 | -2.28 |
| 38 | 2014 | La Pérouse Bay | 58.71823 | -93.88931 | F | AHY | Primary flight | -125.05 | 7.30 | -24.35 | -0.41 |
| 39 | 2014 | La Pérouse Bay | 58.71823 | -93.88931 | F | AHY | Primary flight | -112.87 | 8.34 | -24.90 | 2.22 |
| 40 | 2014 | La Pérouse Bay | 58.71823 | -93.88931 | F | AHY | Primary flight | -126.12 | 6.43 | -24.79 | 0.39 |
| 41 | 2014 | La Pérouse Bay | 58.71823 | -93.88931 | F | AHY | Primary flight | -132.74 | 6.11 | -24.36 | -0.04 |
| 42 | 2014 | La Pérouse Bay | 58.71823 | -93.88931 | F | AHY | Primary flight | -135.31 | 5.97 | -24.11 | -2.92 |
| 43 | 2014 | La Pérouse Bay | 58.71823 | -93.88931 | F | AHY | Primary flight | -135.64 | 5.49 | -23.40 | 0.52 |
| 44 | 2014 | La Pérouse Bay | 58.71823 | -93.88931 | F | AHY | Primary flight | -131.90 | 6.09 | -24.83 | 4.15 |
| 45 | 2014 | La Pérouse Bay | 58.71823 | -93.88931 | F | AHY | Primary flight | -144.90 | 6.88 | -24.15 | 1.40 |
| 46 | 2014 | La Pérouse Bay | 58.71823 | -93.88931 | F | AHY | Primary flight | -125.32 | 8.25 | -24.22 | 4.64 |
| 47 | 2014 | La Pérouse Bay | 58.71823 | -93.88931 | F | AHY | Primary flight | -131.95 | 5.06 | -23.96 | 3.63 |
| 48 | 2014 | La Pérouse Bay | 58.71823 | -93.88931 | F | AHY | Primary flight | -129.32 | 6.10 | -24.22 | 3.92 |
| 49 | 2014 | Baffin Island | 66.71279 | -72.55731 | F | AHY | Primary flight | -151.73 | 7.22 | -24.33 | 4.62 |
| 50 | 2014 | Baffin Island | 66.71279 | -72.55731 | F | AHY | Primary flight | -161.54 | 6.37 | -25.35 | 4.44 |
| 51 | 2014 | Baffin Island | 66.71279 | -72.55731 | F | AHY | Primary flight | -158.35 | 8.01 | -23.49 | 11.02 |
| 52 | 2014 | Baffin Island | 66.71279 | -72.55731 | F | AHY | Primary flight | -161.79 | 6.92 | -24.91 | 1.51 |
| 53 | 2014 | Baffin Island | 66.71279 | -72.55731 | F | AHY | Primary flight | -162.77 | 6.54 | -24.61 | 6.67 |
| 54 | 2014 | Baffin Island | 66.71279 | -72.55731 | F | AHY | Primary flight | -163.91 | 6.05 | -24.08 | 7.61 |
| 55 | 2014 | Baffin Island | 66.71279 | -72.55731 | F | AHY | Primary flight | -161.64 | 7.23 | -23.30 | 1.42 |
| 56 | 2014 | Baffin Island | 66.71279 | -72.55731 | F | AHY | Primary flight | -173.10 | 6.36 | -23.82 | 5.68 |
| 57 | 2016 | Akimiski Island | 53.19662 | -81.55124 | M | AHY | Secondary covert | -108.16 | . | . | . |
| 58 | 2016 | Akimiski Island | 53.19662 | -81.55124 | M | AHY | Secondary covert | -121.12 | . | . | . |
| 59 | 2016 | Akimiski Island | 53.19662 | -81.55124 | M | AHY | Secondary covert | -123.13 | . | . | . |
| 60 | 2016 | Akimiski Island | 53.19662 | -81.55124 | M | AHY | Secondary covert | -115.34 | . | . | . |
| 61 | 2016 | Akimiski Island | 53.19662 | -81.55124 | M | AHY | Secondary covert | -116.80 | . | . | . |
| 62 | 2016 | Akimiski Island | 53.19662 | -81.55124 | M | AHY | Secondary covert | -124.64 | . | . | . |
| 63 | 2016 | Akimiski Island | 53.19662 | -81.55124 | M | AHY | Secondary covert | -118.77 | . | . | . |
| 64 | 2016 | Akimiski Island | 53.19662 | -81.55124 | M | AHY | Secondary covert | -118.89 | . | . | . |
| 65 | 2016 | Akimiski Island | 53.19662 | -81.55124 | M | AHY | Secondary covert | -112.71 | . | . | . |
| 66 | 2016 | La Pérouse Bay | 58.71823 | -93.88931 | F | AHY | Secondary covert | -129.91 | . | . | . |
| 67 | 2016 | La Pérouse Bay | 58.71823 | -93.88931 | F | AHY | Secondary covert | -126.46 | . | . | . |
| 68 | 2016 | La Pérouse Bay | 58.71823 | -93.88931 | F | AHY | Secondary covert | -128.40 | . | . | . |
| 69 | 2016 | La Pérouse Bay | 58.71823 | -93.88931 | M | AHY | Secondary covert | -126.53 | . | . | . |
| 70 | 2016 | La Pérouse Bay | 58.71823 | -93.88931 | F | AHY | Secondary covert | -122.09 | . | . | . |
| 71 | 2016 | La Pérouse Bay | 58.71823 | -93.88931 | F | AHY | Secondary covert | -128.97 | . | . | . |
| 72 | 2016 | La Pérouse Bay | 58.71823 | -93.88931 | M | AHY | Secondary covert | -127.22 | . | . | . |
| 73 | 2016 | La Pérouse Bay | 58.71823 | -93.88931 | M | AHY | Secondary covert | -130.09 | . | . | . |
| 74 | 2016 | La Pérouse Bay | 58.71823 | -93.88931 | F | AHY | Secondary covert | -129.56 | . | . | . |
| 75 | 2016 | La Pérouse Bay | 58.71823 | -93.88931 | F | AHY | Secondary covert | -129.80 | . | . | . |
| 76 | 2016 | Baffin Island | 66.71279 | -72.55731 | F | AHY | Secondary covert | -156.60 | . | . | . |
| 77 | 2016 | Baffin Island | 66.71279 | -72.55731 | M | AHY | Secondary covert | -163.35 | . | . | . |
| 78 | 2016 | Baffin Island | 66.71279 | -72.55731 | M | AHY | Secondary covert | -157.65 | . | . | . |
| 79 | 2016 | Baffin Island | 66.71279 | -72.55731 | F | AHY | Secondary covert | -188.33 | . | . | . |
| 80 | 2016 | Baffin Island | 66.71279 | -72.55731 | M | AHY | Secondary covert | -190.76 | . | . | . |
| 81 | 2016 | Baffin Island | 66.71279 | -72.55731 | M | AHY | Secondary covert | -175.86 | . | . | . |
| 82 | 2016 | Baffin Island | 66.71279 | -72.55731 | F | AHY | Secondary covert | -169.10 | . | . | . |
| 83 | 2016 | Baffin Island | 66.71279 | -72.55731 | M | AHY | Secondary covert | -161.21 | . | . | . |
| 84 | 2016 | Baffin Island | 66.71279 | -72.55731 | F | AHY | Secondary covert | -161.45 |  |  |  |
| 85 | 2016 | Baffin Island | 66.71279 | -72.55731 | F | AHY | Secondary covert | -159.32 |  |  |  |
| 86 | 2016 | Baffin Island | 66.71279 | -72.55731 | F | AHY | Secondary covert | -162.46 |  |  |  |
| 87 | 2016 | Southampton Island | 63.80852 | -85.69742 | M | AHY | Secondary covert | -152.74 | . | . | . |
| 88 | 2016 | Southampton Island | 63.80852 | -85.69742 | M | AHY | Secondary covert | -156.34 | . | . | . |
| 89 | 2016 | Southampton Island | 63.80852 | -85.69742 | F | AHY | Secondary covert | -154.60 | . | . | . |
| 90 | 2016 | Southampton Island | 63.80852 | -85.69742 | F | AHY | Secondary covert | -160.15 | . | . | . |
| 91 | 2016 | Southampton Island | 63.80852 | -85.69742 | M | AHY | Secondary covert | -156.21 | . | . | . |
| 92 | 2016 | Southampton Island | 63.80852 | -85.69742 | F | AHY | Secondary covert | -145.88 | . | . | . |
| 93 | 2016 | Southampton Island | 63.80852 | -85.69742 | M | AHY | Secondary covert | -149.19 | . | . | . |
| 94 | 2016 | Southampton Island | 63.80852 | -85.69742 | M | AHY | Secondary covert | -163.95 | . | . | . |
| 95 | 2016 | Southampton Island | 63.80852 | -85.69742 | F | AHY | Secondary covert | -161.00 | . | . | . |
| 96 | 2016 | Southampton Island | 63.80852 | -85.69742 | M | AHY | Secondary covert | -163.99 | . | . | . |
| 97 | 2016 | Southampton Island | 63.80852 | -85.69742 | M | AHY | Secondary covert | -154.74 | . | . | . |
| 98 | 2016 | Southampton Island | 63.80852 | -85.69742 | F | AHY | Secondary covert | -166.19 | . | . | . |
| 99 | 2016 | Southampton Island | 63.80852 | -85.69742 | F | AHY | Secondary covert | -163.26 | . | . | . |
