# Supplementary material for: A multi-isotope (δ13C, δ15N, δ34S, δ2H) approach to establishing migratory connectivity in lesser snow geese: Tracking an overabundant species
Source: PLoS One. 2018 Aug 24;13(8):e0203077. doi: 10.1371/journal.pone.0203077 (PMC6108521; doi:10.1371/journal.pone.0203077)
Supplement: S2 Dataset — Stable isotope values are reported in per mil notation (‰). (DOCX) [file pone.0203077.s002.docx]

| Individual | Collection Year | Collection State | Sex | Age | Feather Type | δ^2^H |
| --- | --- | --- | --- | --- | --- | --- |
| 1 | 2015 | Arkansas | F | AHY | Primary flight | -153.85 |
| 2 | 2015 | Arkansas | F | AHY | Primary flight | -181.39 |
| 3 | 2015 | Arkansas | F | AHY | Primary flight | -185.66 |
| 4 | 2015 | Arkansas | F | AHY | Primary flight | -163.05 |
| 5 | 2015 | Arkansas | F | AHY | Primary flight | -190.48 |
| 6 | 2015 | Arkansas | F | AHY | Primary flight | -170.84 |
| 7 | 2015 | Arkansas | F | AHY | Primary flight | -168.02 |
| 8 | 2015 | Arkansas | F | AHY | Primary flight | -189.26 |
| 9 | 2015 | Arkansas | F | AHY | Primary flight | -177.57 |
| 10 | 2015 | Arkansas | F | AHY | Primary flight | -173.90 |
| 11 | 2015 | Arkansas | F | AHY | Primary flight | -166.10 |
| 12 | 2015 | Missouri | F | AHY | Primary flight | -156.17 |
| 13 | 2015 | Missouri | F | AHY | Primary flight | -155.10 |
| 14 | 2015 | Missouri | F | AHY | Primary flight | -168.93 |
| 15 | 2015 | Missouri | F | AHY | Primary flight | -182.60 |
| 16 | 2015 | Missouri | F | AHY | Primary flight | -180.70 |
| 17 | 2015 | Missouri | F | AHY | Primary flight | -177.70 |
| 18 | 2015 | Missouri | F | AHY | Primary flight | -164.20 |
| 19 | 2015 | Missouri | F | AHY | Primary flight | -160.90 |
| 20 | 2015 | Missouri | F | AHY | Primary flight | -173.50 |
| 21 | 2015 | Missouri | F | AHY | Primary flight | -180.09 |
| 22 | 2015 | Nebraska | F | AHY | Primary flight | -136.46 |
| 23 | 2015 | Nebraska | F | AHY | Primary flight | -171.07 |
| 24 | 2015 | Nebraska | F | AHY | Primary flight | -148.14 |
| 25 | 2015 | Nebraska | F | AHY | Primary flight | -179.48 |
| 26 | 2015 | Nebraska | F | AHY | Primary flight | -171.55 |
| 27 | 2015 | Nebraska | F | AHY | Primary flight | -185.57 |
| 28 | 2015 | Nebraska | F | AHY | Primary flight | -168.84 |
| 29 | 2015 | Nebraska | F | AHY | Primary flight | -180.34 |
| 30 | 2015 | Nebraska | F | AHY | Primary flight | -156.50 |
| 31 | 2015 | Nebraska | F | AHY | Primary flight | -161.49 |
| 32 | 2015 | Nebraska | F | AHY | Primary flight | -173.78 |
| 33 | 2015 | South Dakota | F | AHY | Primary flight | -181.39 |
| 34 | 2015 | South Dakota | F | AHY | Primary flight | -175.43 |
| 35 | 2015 | South Dakota | F | AHY | Primary flight | -159.49 |
| 36 | 2015 | South Dakota | F | AHY | Primary flight | -169.39 |
| 37 | 2015 | South Dakota | F | AHY | Primary flight | -171.53 |
| 38 | 2015 | South Dakota | F | AHY | Primary flight | -170.31 |
| 39 | 2015 | South Dakota | F | AHY | Primary flight | -171.91 |
| 40 | 2015 | South Dakota | F | AHY | Primary flight | -172.22 |
