# Supplementary material for: A multi-isotope (δ13C, δ15N, δ34S, δ2H) approach to establishing migratory connectivity in lesser snow geese: Tracking an overabundant species
Source: PLoS One. 2018 Aug 24;13(8):e0203077. doi: 10.1371/journal.pone.0203077 (PMC6108521; doi:10.1371/journal.pone.0203077)
Supplement: S1 Table — Percentages in bold identify the greatest probability of colony and subpopulation association for each collected specimen. (PDF) [file pone.0203077.s003.pdf]

S1 - Table

	Colony Association					Subregion Association				Subpopulation Association	
Collected Specimen	**Akimiski Island**	**Baffin Island**	**Karrak Lake**	**La Pérouse Bay**	**Southampton Island**	**Akimiski Island**	**Karrak Lake**	**La Pérouse Bay**	**Baffin / Southampton Islands**	**Arctic**	**Subarctic**
1	0.0%	41.6%	0.4%	5.5%	**52.5%**	0.0%	1.0%	11.4%	**87.6%**	**84.9%**	15.1%
2	0.0%	13.8%	**79.4%**	0.0%	6.8%	0.0%	**90.3%**	0.0%	9.7%	**99.9%**	0.1%
3	0.0%	5.9%	**91.5%**	0.0%	2.6%	0.0%	**96.2%**	0.0%	3.8%	**100.0%**	0.0%
4	0.0%	**49.9%**	4.5%	0.3%	45.3%	0.0%	10.8%	1.0%	**88.2%**	**97.2%**	2.8%
5	0.0%	2.1%	**97.1%**	0.0%	0.8%	0.0%	**98.8%**	0.0%	1.2%	**100.0%**	0.0%
6	0.0%	**45.0%**	23.3%	0.0%	31.6%	0.0%	43.0%	0.1%	**56.9%**	**99.3%**	0.7%
7	0.0%	**48.9%**	13.4%	0.1%	37.7%	0.0%	28.0%	0.3%	**71.7%**	**98.8%**	1.2%
8	0.0%	2.8%	**96.2%**	0.0%	1.0%	0.0%	**98.4%**	0.0%	1.6%	**100.0%**	0.0%
9	0.0%	25.0%	**60.9%**	0.0%	14.0%	0.0%	**79.1%**	0.0%	20.9%	**99.8%**	0.2%
10	0.0%	37.3%	**39.1%**	0.0%	23.6%	0.0%	**61.1%**	0.0%	38.8%	**99.6%**	0.4%
11	0.0%	**50.0%**	8.9%	0.1%	41.0%	0.0%	19.9%	0.5%	**79.7%**	**98.3%**	1.7%
12	0.0%	45.2%	0.8%	2.4%	**51.5%**	0.0%	2.2%	5.7%	**92.1%**	**90.8%**	9.2%
13	0.0%	44.1%	0.6%	3.2%	**52.1%**	0.0%	1.7%	7.3%	**91.0%**	**89.0%**	11.0%
14	0.0%	**48.0%**	16.1%	0.1%	35.9%	0.0%	32.5%	0.2%	**67.3%**	**99.0%**	1.0%
15	0.0%	11.0%	**83.7%**	0.0%	5.2%	0.0%	**92.5%**	0.0%	7.5%	**99.9%**	0.1%
16	0.0%	15.5%	**76.6%**	0.0%	7.9%	0.0%	**88.7%**	0.0%	11.3%	**99.9%**	0.1%
17	0.0%	24.7%	**61.5%**	0.0%	13.8%	0.0%	**79.4%**	0.0%	20.5%	**99.8%**	0.2%
18	0.0%	**50.2%**	5.8%	0.2%	43.8%	0.0%	13.6%	0.8%	**85.6%**	**97.7%**	2.3%
19	0.0%	**49.0%**	2.7%	0.6%	47.7%	0.0%	6.7%	1.8%	**91.5%**	**95.8%**	4.2%
20	0.0%	**38.5%**	36.8%	0.0%	24.7%	0.0%	**58.9%**	0.0%	41.1%	**99.6%**	0.4%
21	0.0%	17.2%	**73.9%**	0.0%	8.9%	0.0%	**87.2%**	0.0%	12.8%	**99.9%**	0.1%
22	0.7%	4.5%	0.0%	**84.9%**	9.9%	1.0%	0.0%	**86.6%**	12.3%	21.7%	**78.3%**
23	0.0%	**44.4%**	24.6%	0.0%	30.9%	0.0%	44.8%	0.1%	**55.1%**	**99.3%**	0.7%
24	0.0%	32.4%	0.1%	19.1%	**48.3%**	0.0%	0.2%	30.1%	**69.6%**	**69.4%**	30.6%
25	0.0%	19.0%	**71.1%**	0.0%	10.0%	0.0%	**85.5%**	0.0%	14.5%	**99.9%**	0.1%
26	0.0%	**43.6%**	26.4%	0.0%	29.9%	0.0%	47.1%	0.1%	**52.8%**	**99.4%**	0.6%
27	0.0%	6.1%	**91.3%**	0.0%	2.6%	0.0%	**96.2%**	0.0%	3.8%	**100.0%**	0.0%
28	0.0%	**48.1%**	15.8%	0.1%	36.1%	0.0%	32.0%	0.2%	**67.8%**	**99.0%**	1.0%
29	0.0%	16.6%	**74.8%**	0.0%	8.5%	0.0%	**87.7%**	0.0%	12.3%	**99.9%**	0.1%
30	0.0%	45.5%	0.9%	2.2%	**51.4%**	0.0%	2.4%	5.3%	**92.3%**	**91.2%**	8.8%
31	0.0%	**49.3%**	3.1%	0.5%	47.1%	0.0%	7.6%	1.6%	**90.8%**	**96.2%**	3.8%
32	0.0%	37.6%	**38.5%**	0.0%	23.9%	0.0%	**60.6%**	0.0%	39.4%	**99.6%**	0.4%
33	0.0%	13.8%	**79.4%**	0.0%	6.8%	0.0%	**90.3%**	0.0%	9.7%	**99.9%**	0.1%
34	0.0%	32.5%	**47.9%**	0.0%	19.6%	0.0%	**69.2%**	0.0%	30.8%	**99.7%**	0.3%
35	0.0%	48.1%	1.9%	0.9%	**49.1%**	0.0%	4.9%	2.6%	**92.6%**	**94.7%**	5.3%
36	0.0%	**47.3%**	17.8%	0.0%	34.8%	0.0%	35.2%	0.2%	**64.7%**	**99.1%**	0.9%
37	0.0%	**43.6%**	26.4%	0.0%	29.9%	0.0%	47.1%	0.1%	**52.8%**	**99.4%**	0.6%
38	0.0%	**45.9%**	21.2%	0.0%	32.8%	0.0%	40.2%	0.1%	**59.7%**	**99.2%**	0.8%
39	0.0%	**42.7%**	28.4%	0.0%	28.9%	0.0%	49.5%	0.1%	**50.4%**	**99.4%**	0.6%
40	0.0%	**42.0%**	29.9%	0.0%	28.2%	0.0%	**51.3%**	0.1%	48.7%	**99.4%**	0.6%
